# Network-based pharmacology-based research on the effect and mechanism of the *Hedyotis diffusa*–*Scutellaria Barbata* pair in the treatment of hepatocellular carcinoma

**DOI:** 10.1038/s41598-023-50696-y

**Published:** 2024-01-10

**Authors:** Changmiao Hou, Xiao Wen, Shifan Yan, Xiaoxiao Gu, Yu Jiang, Fang Chen, Yanjuan Liu, Yimin Zhu, Xiehong Liu

**Affiliations:** 1 Hunan Provincial Key Laboratory of Emergency and Critical Care Metabonomics, Institute of Emergency Medicine, Hunan Provincial People’s Hospital (The First Affiliated Hospital of Hunan Normal University), Changsha, Hunan China; 2 Department of Emergency, Hunan Provincial People’s Hospital (The First Affiliated Hospital of Hunan Normal University), Changsha, Hunan China; 3 Hunan University of Traditional Chinese Medicine, Changsha, Hunan China

**Keywords:** Cancer, Molecular biology

## Abstract

The *Hedyotis diffusa*–*Scutellaria officinalis pair* (HD–SB) has therapeutic effects on a variety of cancers. Our study was to explore the mechanism of HD–SB in the treatment of hepatocellular carcinoma (HCC). A total of 217 active ingredients of HD–SB and 1196 HCC-related targets were reserved from the TCMSP and the SwissTarget Prediction database, and we got 63 intersection targets from GeneCards. We used a Venn diagram, and Cytoscape found that the three core ingredients were quercetin, luteolin, and baicalein. The PPI analysis showed that the core targets were TP53, CDK2, XPO1, and APP. Molecular docking results showed that these core ingredients had good binding potential with the core targets. HD–SB acts simultaneously on various HCC-related signaling pathways, including proteoglycans in cancer and the P53 signaling pathway. In vitro experiments confirmed that HD–SB can inhibit HepG2 cell proliferation by increasing TP53 and APP levels and decreasing XPO1 and CDK2 levels. This study analyzed active ingredients, core targets, and central mechanisms of HD–SB in the treatment of HCC. It reveals the role of HD–SB in targeting the P53 signaling pathway in the treatment of HCC. We hope that our research could provide a new perspective to the therapy of HCC and find new anticancer drugs.

## Introduction

Hepatocellular carcinoma (HCC) is a common malignant tumour in the Department of Gastroenterology. According to cancer statistics in 2020, approximately 906,000 people are diagnosed with HCC every year, representing 4.7% of cancer incidence, and about 830,000 people die of HCC, which seriously endangers human life, health, and safety^1^. The proportion of HCC caused by hepatitis B virus infection in China is as high as 92.05%^2^. Therefore, it is particularly important for the prevention and treatment of HCC. Non-pharmacological treatments such as hepatectomy resection, liver transplantation, and transarterial chemoembolization are beneficial for patients with early HCC. However, most patients with HCC have developed to the middle and advanced stage when diagnosed, and targeted drugs and immunocheckpoint inhibitor therapy with PD-1 / PD-L1 respond only in part of the dominant group, and treatment methods are very limited^3,4^. Given the shortcomings mentioned above of current clinical treatment approaches, the addition of Traditional Chinese Medicine (TCM) enhanced the comprehensive antitumor effect and therefore emerges as the focus of HCC treatment^5,6^.

The *Hedyotis diffusa* and *Scutellaria barbata herb pair* (HD–SB) are often used for clinical cancer treatment with a definitive curative effect^7^. *scutellaria barbata* mainly contains flavonoids, while *hedyotis diffusa* mainly contains terpenoids and flavonoids^8^. Compared to the ethanol extract of a single drug, the HD–SB ethanol extract can significantly inhibit the growth of human colon cancer cell lines, indicating that the anticancer effect of the combined use of the two drugs will be enhanced^9^. Additionally, Studies have shown that the polysaccharides extracted from HD–SB inhibited the proliferation and the transformation from G1 phase to S phase of S180 tumor cell line^10^. Similarly, the studies have shown that HD–SB regulated the cell cycle of H22 hepatoma cells, and arrested cells in G1-S phase^11,12^. Importantly, network pharmacology reveals multiple mechanisms of HD–SB in colorectal cancer^13^ and antiovarian cancer^14^. Therefore, Using network pharmacology to further study the mechanism of HD–SB in the treatment of HCC will contribute to improve the therapeutic effect.

**Figure 1:**
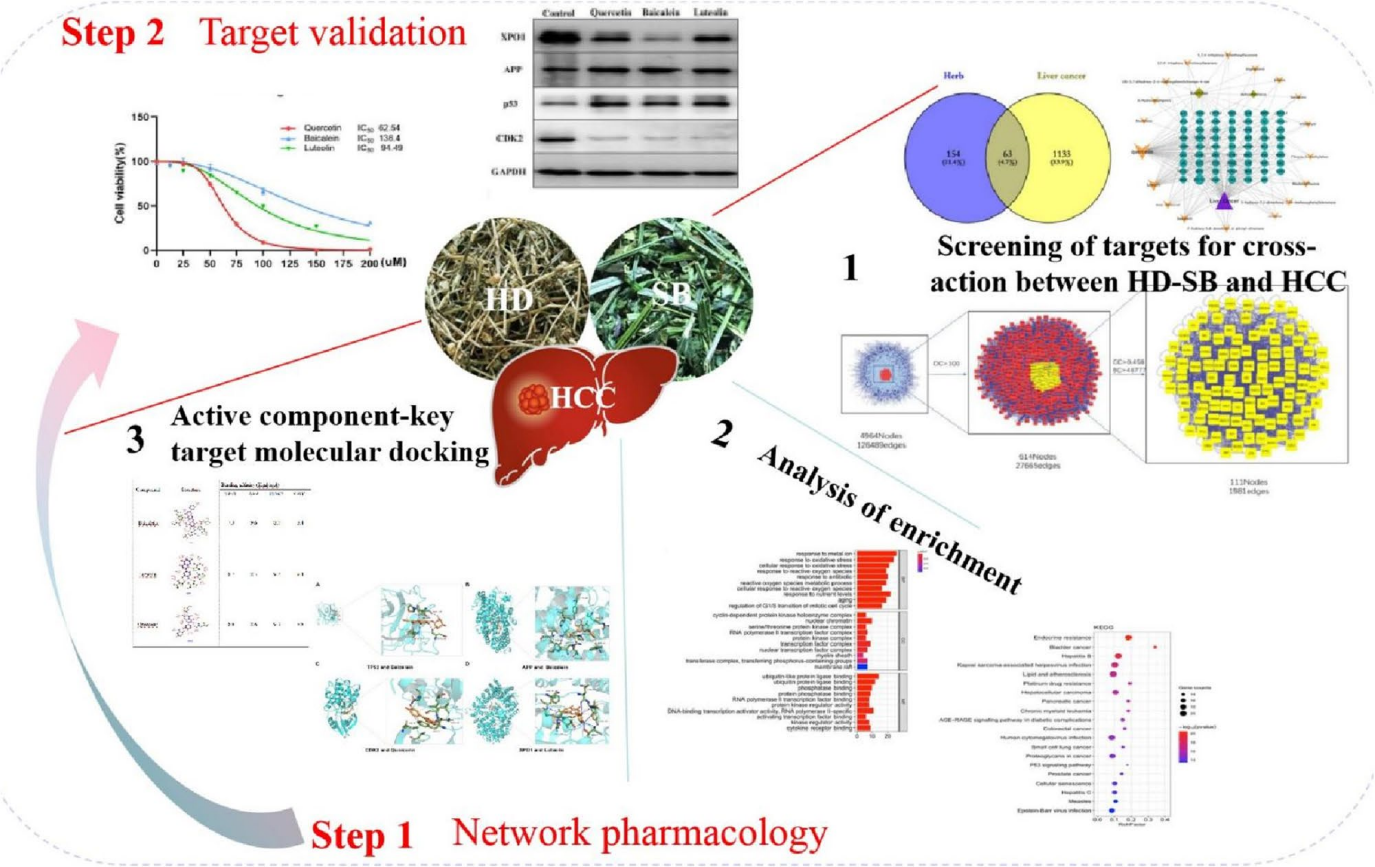
The graphical abstract depicts the the research workflow.

In this present study, network pharmacology was used to analyse active ingredients, potential targets, and main mechanisms of HD–SB in the treatment of HCC, and to construct the herb-ingredient-target-disease network, to provide a reference for the study of the specific mechanism of the drug in the treatment of HCC. The graphical abstract (Fig. 1) depicts the the research workflow.

## Results

### The active ingredients and the corresponding targets of HD–SB

We applied a network-based pharmacology strategy to explore the mechanisms by which HD–SB affects HCC cells. The active components of HD and SB were recovered from the TCMSP. There were 7 species of HD and 31 species of SB. After screening with values of OB (30%) and DL (≥ 0.18) values, 7 species of HD and 29 species of SB were obtained (Table 1). An online search for targets for 36 compounds in the TCMSP and Uniprot database resulted in 217 targets collected.

**Table 1:** The active ingredient in a drug.

Herb	MOL ID	Compound name	OB(%)	DL
Hedyotis diffusa	MOL001646	2,3-dimethoxy-6-methyanthraquinone	34.85860475	0.26255
	MOL001659	Poriferasterol	43.82985158	0.75596
	MOL001663	(4aS,6aR,6aS,6bR,8aR,10R,12aR,14bS)-10-hydroxy-2,2,6a,6b,9,9,12a-heptamethyl-1,3,4,5,6,6a,7,8,8a,10,11,12,13,14b-tetradecahydropicene-4a-carboxylic acid	32.02801329	0.75713
	MOL001670	2-Methoxy-3-methyl-9,10-anthraquinone	37.82770556	0.20517
	MOL000449	Stigmasterol	43.82985158	0.75665
	MOL000358	Beta-sitosterol	36.91390583	0.75123
	MOL000098	Quercetin	46.43334812	0.27525
Sculellaria barbata	MOL001040	(2R)-5,7-dihydroxy-2-(4-hydroxyphenyl)chroman-4-one	42.36332114	0.21141
	MOL012245	5,7,4′-trihydroxy-6-methoxyflavanone	36.62688628	0.26833
	MOL012246	5,7,4′-Trihydroxy-8-methoxyflavanone	74.23522001	0.26479
	MOL012248	5-Hydroxy-7,8-dimethoxy-2-(4-methoxyphenyl)chromone	65.81880606	0.32874
	MOL012250	7-Hydroxy-5,8-dimethoxy-2-phenyl-chromone	43.7169646	0.25376
	MOL012251	Chrysin-5-methylether	37.2683358	0.20317
	MOL012252	9,19-cyclolanost-24-en-3-ol	38.68565906	0.78074
	MOL002776	Baicalin	40.12360996	0.75264
	MOL012254	Campesterol	37.57681789	0.71486
	MOL000953	CLR	37.87389754	0.67677
	MOL000358	Beta-sitosterol	36.91390583	0.75123
	MOL012266	Rivularin	37.94023355	0.3663
	MOL001973	Sitosteryl acetate	40.38964165	0.85102
	MOL012269	Stigmasta-5,22-dien-3-ol-acetate	46.44190225	0.85814
	MOL012270	Stigmastan-3,5,22-triene	45.02668769	0.71047
	MOL000449	STIGMASTEROL	43.82985158	0.75665
	MOL000173	Wogonin	30.68456706	0.22942
	MOL001735	Dinatin	30.97205344	0.27025
	MOL001755	24-Ethylcholest-4-en-3-one	36.08361164	0.75703
	MOL002714	Baicalein	33.51891869	0.20888
	MOL002719	6-Hydroxynaringenin	33.22920875	0.24203
	MOL002915	Salvigenin	49.06592606	0.33279
	MOL000351	Rhamnazin	47.14113124	0.33648
	MOL000359	Sitosterol	36.91390583	0.7512
	MOL005190	Eriodictyol	71.7926526	0.24372
	MOL005869	Daucostero_qt	36.91390583	0.75177
	MOL000006	Luteolin	36.16262934	0.24552
	MOL008206	Moslosooflavone	44.08795959	0.25331
	MOL000098	Quercetin	46.43334812	0.27525

### Target data analysis of “HD–SB–HCC”

**Figure 2:**
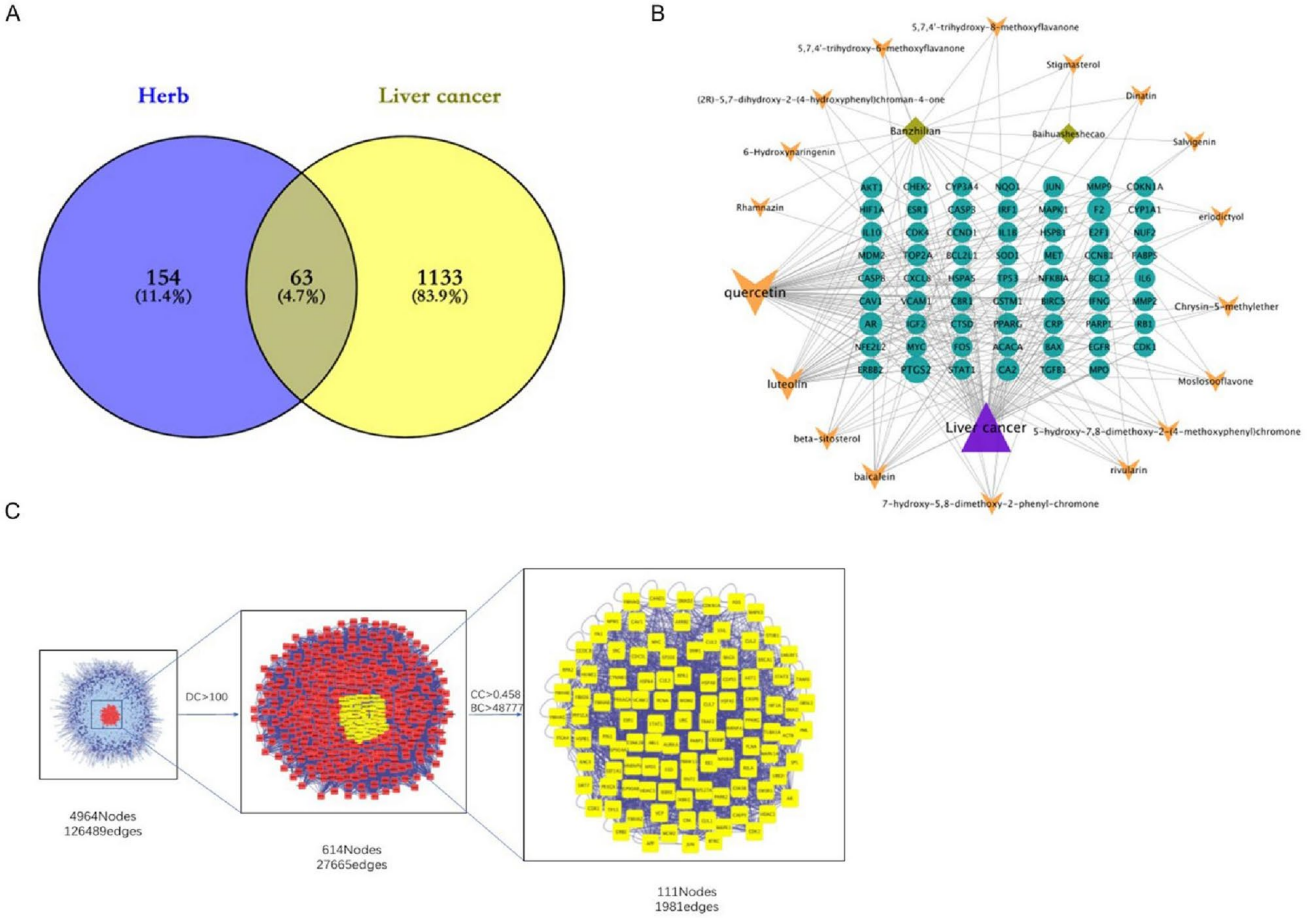
Screening of targets for cross-action between HD–SB and HCC. (**A**) 217 HD–SB targets intersected 1196 HCC targets for 63 common targets. (**B**) Relationship between the active ingredients of HD–SB and the common targets of HD–SB and HCC. (**C**) Get the protein–protein interaction (PPI) network from online STRING.

The target data of "HD–SB–HCC" were selected and analysed by GeneCards databases, and the relevance score of the screening condition was established < 15, and finally 1196 qualified targets were obtained. Through the Venn diagram, the target crossover of "HD–SB–HCC" was performed and 63 targets were obtained (Fig. 2A). These 63 targets are potentially effective targets for HD–SB for HCC, which is the main target of our next study.

### Visual analysis of the targets of HD–SB–HCC

Cytoscape 3.8.2 was used to analyse the data of 63 common drugs and diseases targets, and the interaction diagram of the “drug compounds and disease targets” was obtained (Fig. 2B). It contained 18 active ingredient nodes, 62 target nodes, and 225 edges. The size of the node is set according to its degree value, and the larger the node, the larger its degree value. The network graph is analysed for topological parameters, where the degree value of the node is a key indicator of the importance of the nodes, the average degree value of the network graph is 9.1111 and the top three compounds in the degree value ranking were Quercetin, Luteolin, and Baicalein. The top compounds may be critical nodes in the network and possess an important anti-HCC effect.

On the basis of the target protein interaction data obtained from the String database, the protein interaction network analysis diagram was obtained. Then the Bisogenet plug-in of Cytoscape3.8.2 software is used to analyse the PPI network interaction of 63 key targets, and a network graph with 4964 nodes and 126,489 edges is obtained. Then the topology parameters (DC, BC and CC) of the network graph are analyzed by the CytoNCA plug-in, and the mean value of DC > 2 times (100) is selected as the filtering condition to obtain the preliminary core target network graph, the network graph consisting of 614 nodes and 27,665 edges. Finally, a network graph of 111 nodes and 1981 edges with CC > 0.458 and BC > 48,777 with CC > 0.458 and BC > 48,777 was obtained with CC > 0.458 and BC > 48,777 as screening conditions for the HD–SB and HCC target network target network (Fig. 2C). As shown in Table 2, based on the 111 targets obtained from the topological analysis, a total of 15 core genes were obtained based on the condition of greater than the average value of DC, BC and CC, which were considered as possible key targets for the treatment of HCC.

**Table 2:** Information of 15 core targets in topological analysis.

DC	BC	CC	Gene
947	1,204,758	0.538	**TP53**
848	1,220,727	0.532	**APP**
793	423,543	0.522	CUL3
763	713,692	0.531	ESR1
681	508,968	0.518	**XPO1**
662	298,221	0.511	MCM2
629	348,499	0.51	FN1
615	391,646	0.521	UBC
596	539,186	0.509	MYC
567	225,987	0.503	**CDK2**
545	199,824	0.508	COPS5
536	338,340	0.521	HSP90AA1
530	174,515	0.496	RNF2
475	302,933	0.502	GRB2
465	189,006	0.508	YWHAZ

Significance values are in bold.

### Visualisation of enrichment analysis

**Figure 3:**
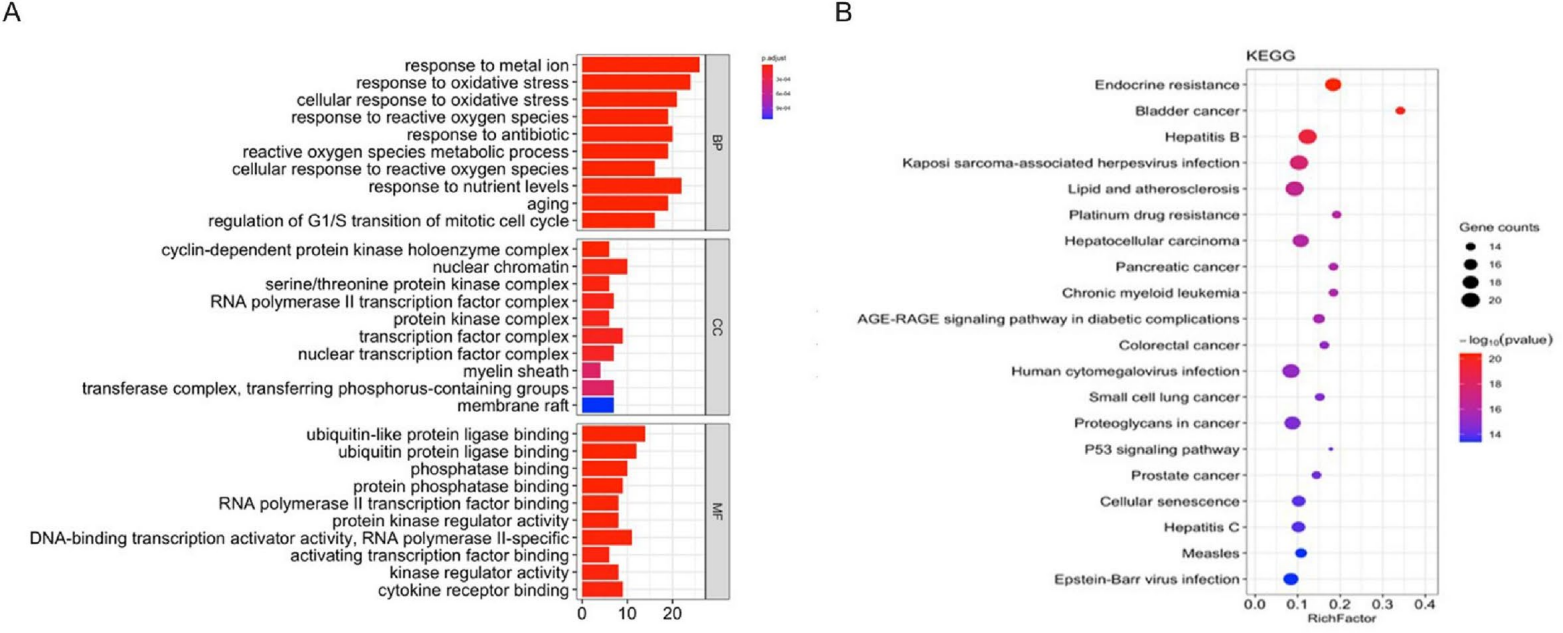
Analysis of enrichment. (**A**) The top ten for GO–BP, GO–CC, and GO–MF enrichment. (**B**) Twenty KEGG pathways that were highly correlated with HCC research.

GO enrichment analysis and the KEGG pathway enrichment analysis^15^ were performed to elucidate the function and enrichment pathways of HD–SB for HCC. GO functional enrichment analysis was performed on 63 potential targets through the DAVID database with *p* < 0.05 as the screening condition and 1887 items were obtained, including 1456 biological processes (BP) and 24 cell compositions (CC) and 60 molecular functions (MF). The top ten of GO–BP, GO–CC, and GO–MF were selected for visual analysis. As demonstrated in Fig. 3A, MFs include mainly ubiquitin-like protein ligase binding, ubiquitin protein ligase binding, and DNA-binding transcription activator activity. CCs mainly include the regulation of reactive oxygen metabolism, the RNA polymerase II transcription factor complex, and the transcription factor complex. BPs mainly include the oxidative stress response, cell response to oxidative stress, reactive oxygen metabolism, and cell response to external stimuli.

Twenty KEGG pathways that were highly correlated with HCC research and their corresponding targets were selected for visual analysis (Fig. 3B). And the top 20 items mainly involve endocrine resistance, the P53 signaling pathway, cellular senescence, lipids and atherosclerosis, the AGE-RAGE signaling pathway in diabetic complications, and HCC. The results showed that HD–SB acted on multiple signal pathways to treat HCC.

### Active component-key target molecular docking

To further verify whether the three active ingredients of HD–SB could effectively bind to the key target proteins of TP53, CDK2, XPO1 and APP, we performed molecular docking between the target proteins and the active ingredients by AutoDock Vina software. Generally, the binding energy is less than − 4.25 kcal/mol, indicating that the small ligand molecule had certain activity with the receptor protein, less than − 5 kcal/mol indicated good activity, and less than − 7 kcal/mol indicated strong activity. As shown in Table 3, the conformations of active compounds of Quercetin, Luteolin, Baicalein and the main protein targets showed good binding interactions, and the interactions were also reliable.

**Table 3:** Virtual docking of three vital active compounds from HD–SB for hepatocellular carcinoma targets.

Compound	Structure	Binding affinity (Kcal/mol)			
		TP53	APP	CDK2	XPO1
Baicalein		− 7.3	− 9.6	− 8.7	− 8.4
Luteolin		− 6.7	− 8.5	− 9.2	− 9.4
Quercetin		− 6.8	− 8.6	− 9.3	− 8.8

**Figure 4:**
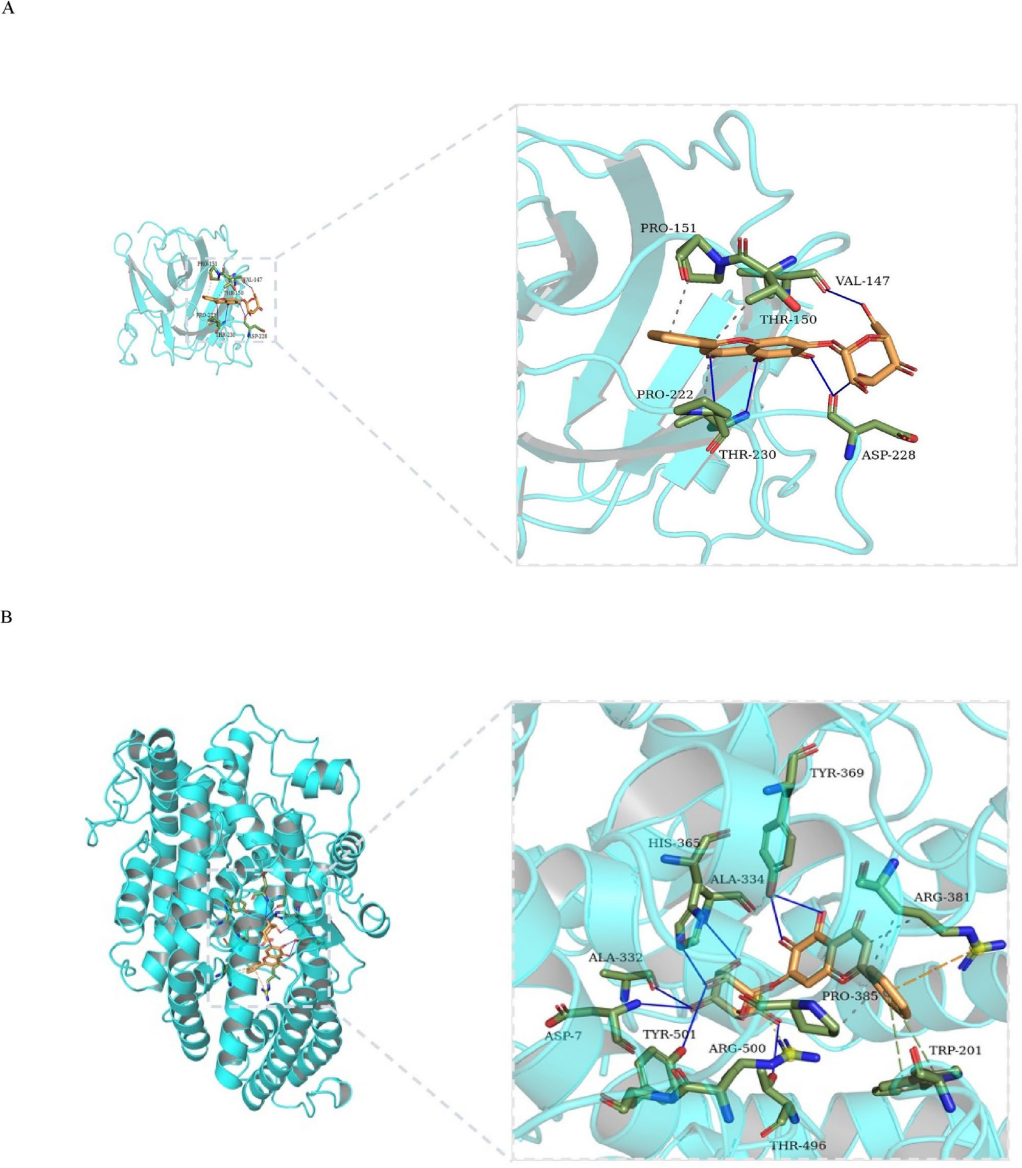

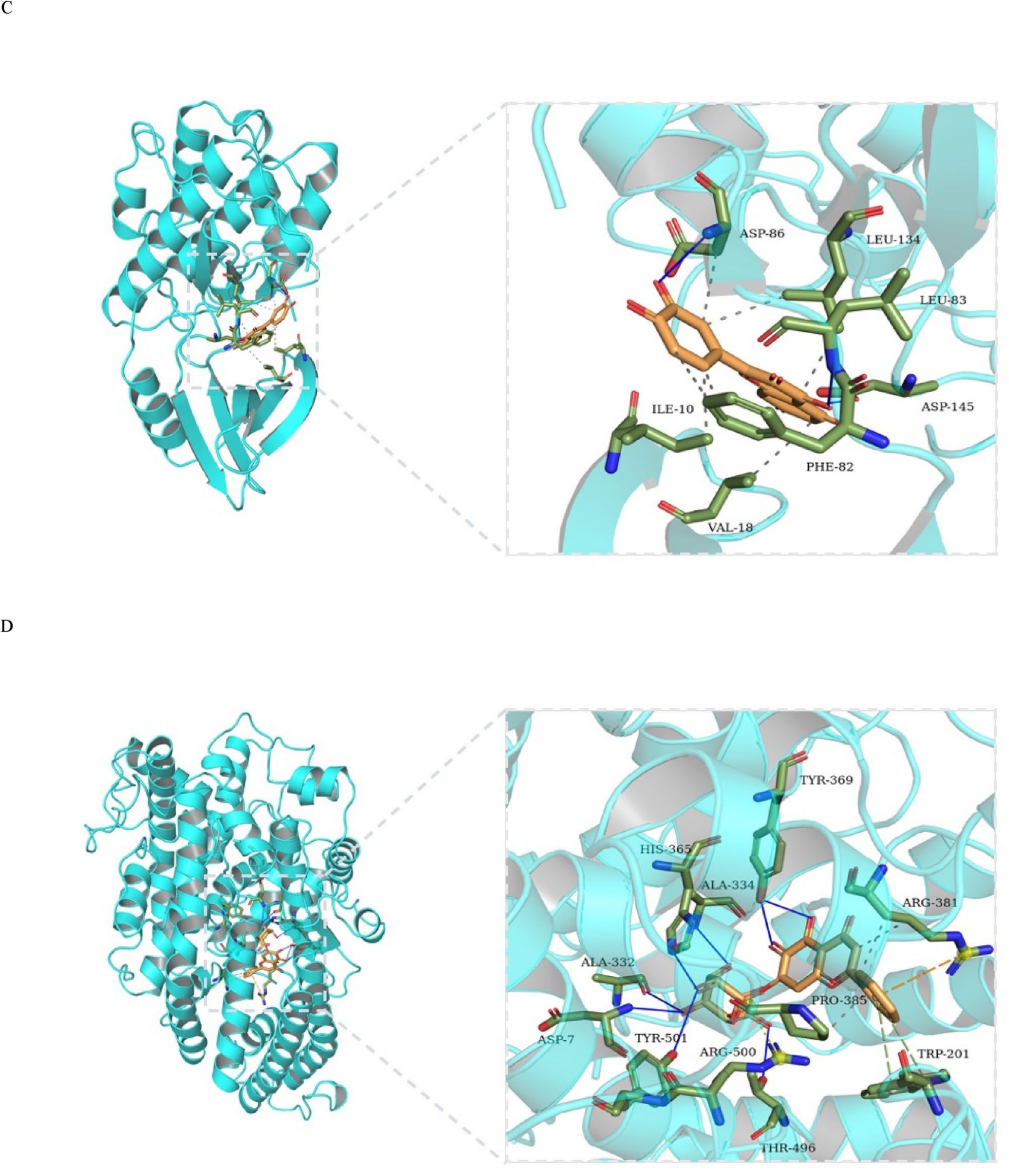
The network of target drug compounds. The docking model of Baicalin, Quercetin and Luteolin with TP53, APP, CDK2 and XPO1, respectively.

The conformations of the minimal binding energies for the key active compounds and the major hub targets are shown in Fig. 4. The analysis showed that P53 and Baicalein formed three hydrogen bonds at Val-147 with bond lengths of 3.12, 2.88 and 3.24, respectively, and formed two hydrogen bonds with bond lengths of 2.75 and 2.89 at Thr-230 and 3.09 and 3.11 at Pro-151, respectively (Fig. 4A). As shown in Fig. 4B, APP and Baicalin formed two hydrogen bonds at Tyr369 with bond lengths of 2.92 and 2.97, respectively. Hydrogen bonds with bond lengths of 3.24, 2.96, 3.16, 2.8 and 2.75 were formed in Thr496, Asp7, Ala334, His365, and Glu389, respectively. As shown in Fig. 4C, CDK2 and quercetin form two hydrogen bonds of 3.28 and 3.08 at Asp145, 3.07 and 2.68 at Leu134, and 2.87 at Asp86. XPO1 and luteolin formed three hydrogen bonds at Val40 with bond lengths of 3.22, 2.74 and 2.92, respectively, and formed hydrogen bonds at Val-18 and Ala-31 with bond lengths of 3.09, 2.89, 3.08 and 2.97, respectively (Fig. 4D). Together, three representative compounds ( Quercetin, Luteolin and Baicalein) of HD–SB could bind well to four core targets of HCC (P53, CDK2, XPO1 and APP), all of which could play key roles in the treatment of HCC.

### Effect of three representative compounds of HD–SB on the proliferation of hepatoma carcinoma cells

**Figure 5:**
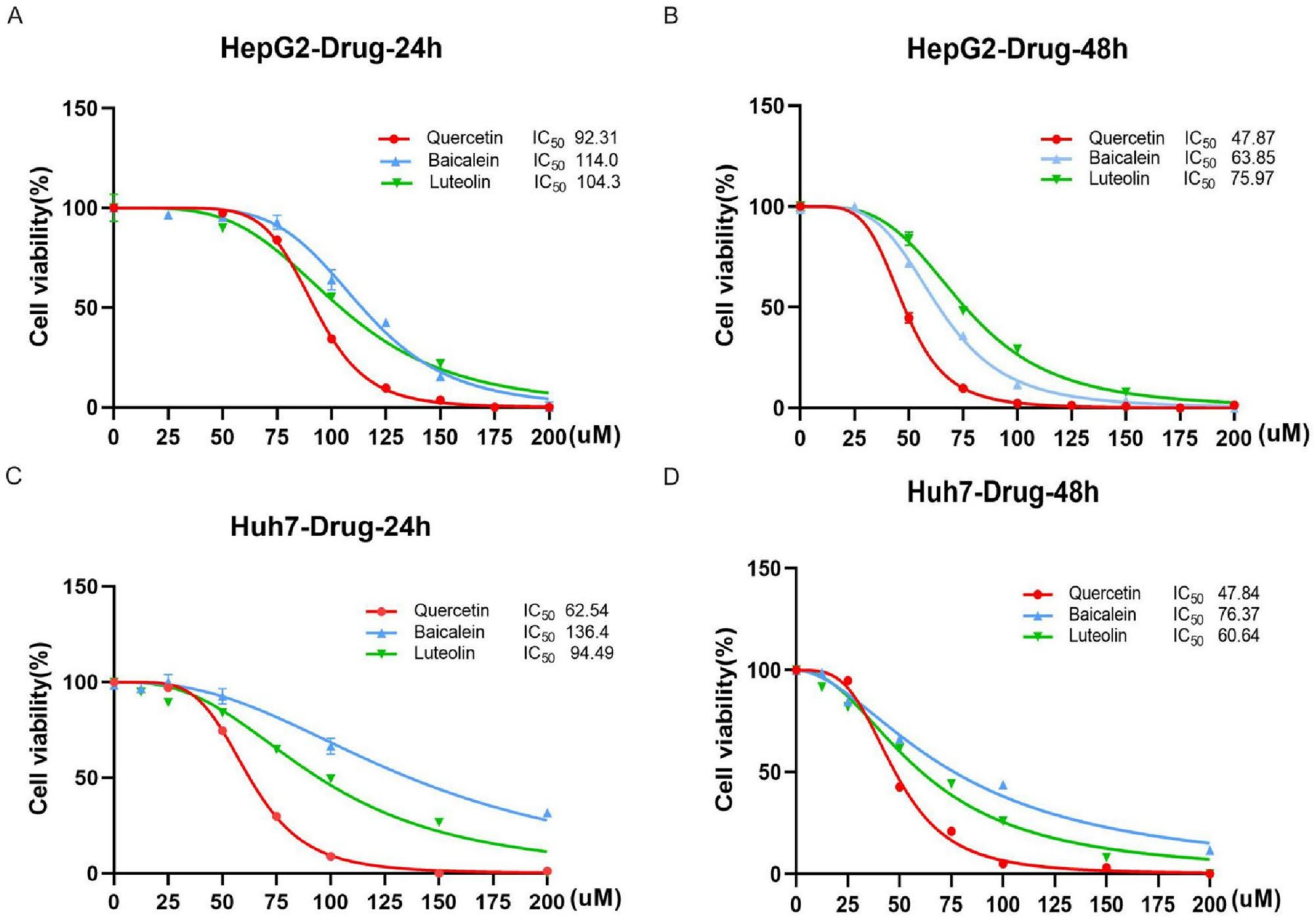
Three representative compounds of HD–SB inhibit the proliferation of hepatoma carcinoma cells. (**A** and **B**) HepG2 cells were treated with the indicated concentrations of Baicalin, Quercetin and Luteolin respectively (0, 25, 50, 75, 100, 125, 175 and 200 μM) for (**A**) 24 h and (**B**) 48 h. CCK-8 assays were used to measure cell viability. (**C** and **D**) Huh7 cells were treated with the indicated concentrations of Baicalin, Quercetin, and Luteolin, respectively (0, 25, 50, 75, 100, 125, 175 and 200 μM) for (**C**) 24 h and (**D**) 48 h. CCK-8 assays were used to measure cell viability. (Error bars represent the S.D. of the mean. n ≥ 6; *p < 0.05;**p < 0.01;***p < 0.001.).

To determine whether the three representative compounds (quercetin, luteolin and baicalein) of HD–SB influence HCC cell proliferation, we first examined the effect of the three pharmaceutical ingredients above on HepG2 cell proliferation with the CCK-8 assay. HepG2 cells were treated with different concentrations of quercetin, luteolin, Baicalein for 24 and 48 h, respectively. Our results showed that Quercetin, Luteolin and Baicalein all decreased cell viability in a dose-dependent and time-dependent manner (Fig. 5A and B). And the same phenomenon was present in Huh7 cells (Fig. 5C and D). These findings suggest that HD–SB suppresses the proliferation of HCC cells.

### Three representative HD–SB compounds modulate the expression of TP53, XPO1, APP, and CDK2 in hepatoma carcinoma cells

**Figure 6:**
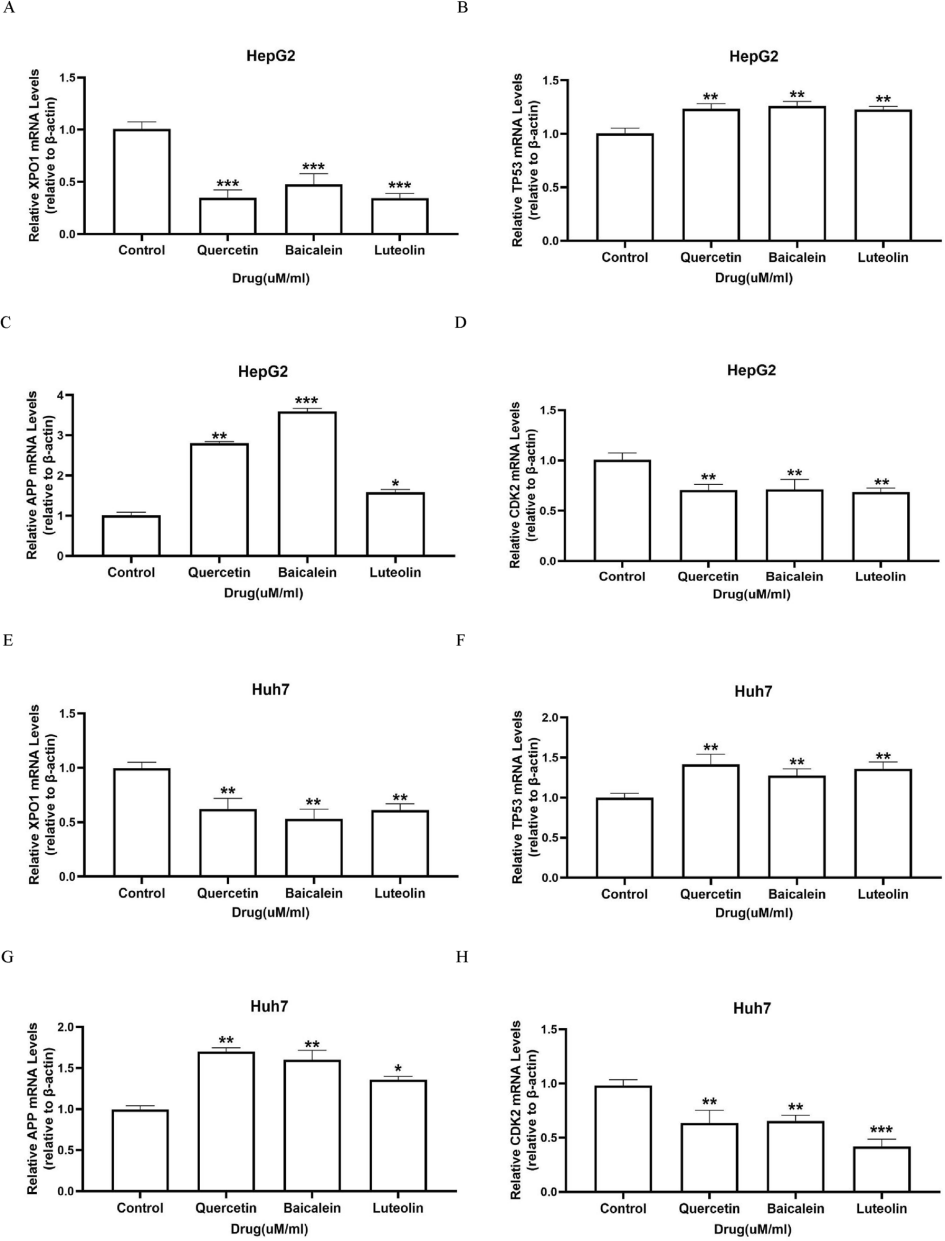
XPO1, TP53, APP and CDK2 are related to the inhibition of hepatoma carcinoma cells induced by three representative HD–SB compounds. HepG2 and Huh7 cells were treated with Baicalin, Quercetin, and Luteolin, respectively, or DMSO for 48 h. (**A**–**D**) Relative expression of XPO1, TP53, APP, and CDK2 mRNA in HepG2 cells compared to the control. (**E**–**H**) Relative expression of XPO1, TP53, APP, and CDK2 mRNA in Huh7 cells compared to the control. Graphs are representatives of three independent experiments (n ≥ 6; *p < 0.05; **p < 0.01; ***p < 0.001).

**Figure 7:**
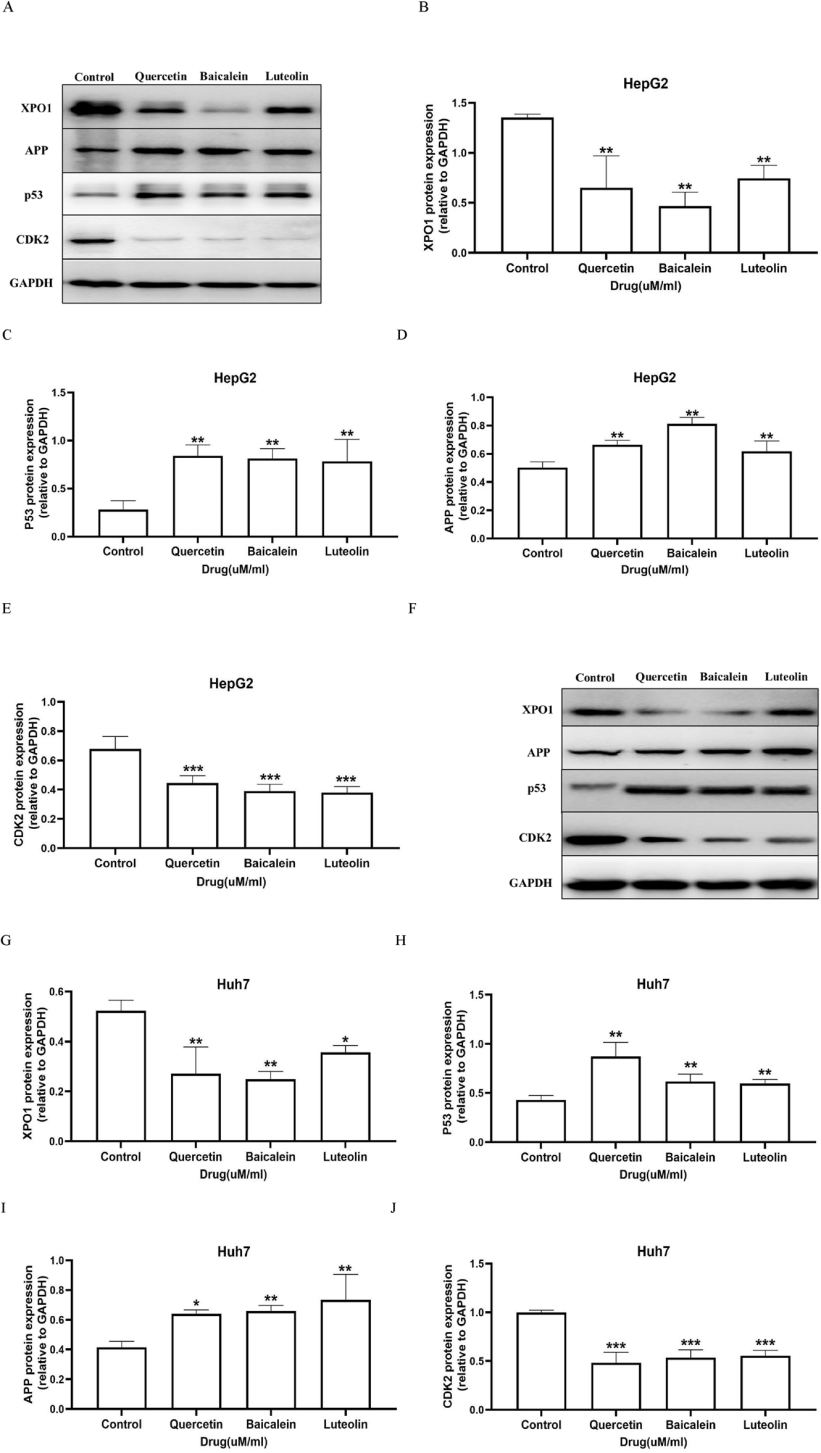
XPO1, TP53, APP, and CDK2 are related to the inhibition of hepatoma carcinoma cells induced by three representative HD–SB compounds. HepG2 and Huh7 cells were treated with Baicalin, Quercetin, and Luteolin, respectively, or DMSO for 48 h. Western blot test of protein levels of XPO1, TP53, APP, and CDK2. Densitometry revealed the fold expression ofXPO1, TP53, APP, and CDK2 compared to that of β-actin, respectively. Graphs are representatives of three independent experiments (n ≥ 6; *p < 0.05; **p < 0.01; ***p < 0.001).

The transcription factor TP53 is an important suppressor of tumour development, which can reduce HCC cell proliferation by inhibiting CDK2 expression^16^. XPO1 is the main mediator of nucleocytoplasmic transport and is overexpressed in a variety of human malignancies and closely related to tumour occurrence and development^17^. APP inhibits apoptosis by mediating apoptosis proteins (Bcl-2)^18^. Bioinformatic analysis has indicated that Quercetin, Baicalein, and Luteolin might play an important role in HCC by regulating the four genes mentioned above, and then we next investigated the effect of three representative compounds on the expression of TP53, XPO1, APP and CDK2 in HepG2 cells and Huh7 cells. The IC50 value at 48 h of drug treatment was selected as our subsequent experiment. Four representative compounds of HD–SB caused a significant decrease in mRNA levels of XPO1 and CDK2 and increase of TP53 and APP, compared with those in control group in HepG2 cells (Fig. 6A–D) and Huh7 cells (Fig. 6E–H). As shown in Fig. 7, Western blots also illustrated that pharmaceutical ingredients significantly decreased XPO1 and CDK2 protein expression and increased TP53 and APP. Taken together, our data further verified the network pharmacological results of the impotent functions of TP53, XPO1, APP, and CDK2 in HD–SB for HCC.

## Discussion

The efficacy of HD–SB is mainly to remove heat and detoxify the toxin and is slightly bitter. In clinical practise, this drug is commonly used in antitumor therapy, and the combination of HD–SB can exert a synergistic effect to promote endometrial cancer apoptosis, breast cancer cells, cervical cancer cells, gastric cancer cells, HCC cells and pancreatic cancer cells and inhibit tumour cell proliferation and differentiation^19,20^. Some experiments have shown that HD–SB has a good tumour suppressor effect in HCC H22-bearing mice^21,22^ and S18 sarcoma mice^23,24^. In this study, a total of 18 active ingredients were identified through network pharmacology, 63 key targets were analysed and 129 pathways related to HCC were involved, reflecting the mechanism of interregulation of HD–SB'multicomponent, multitarget, and multipathways’ to treat HCC.

The studies confirmed that HD–SB could induce apoptosis in HCC cells by increasing the Bax/Bcl-2 ratio^25^. Furthermore, the active ingredients of HD–SB of Quercetin and Baicalein promoted the arrest of the HepG2 cell cycle by upregulating the expression of TP53 and P21^26,27^, and Quercetin and Luteolin inhibited CDK2 activity to arrest the HCC cell cycle^28^. In our study, the analysis of the "TCM compound-target" network revealed that Baicalein, Luteolin, and Quercetin were the active ingredients of HD–SB in the treatment of HCC. The molecular docking results further confirmed that TP53, APP, CDK2 and XPO1 were able to bind to Quercetin, Baicalin and Luteolin and formed stable compounds. In vitro, we verified the network pharmacology results of the impotent functions of TP53, XPO1, APP and CDK2 in HD–SB for HCC.

**Figure 8:**
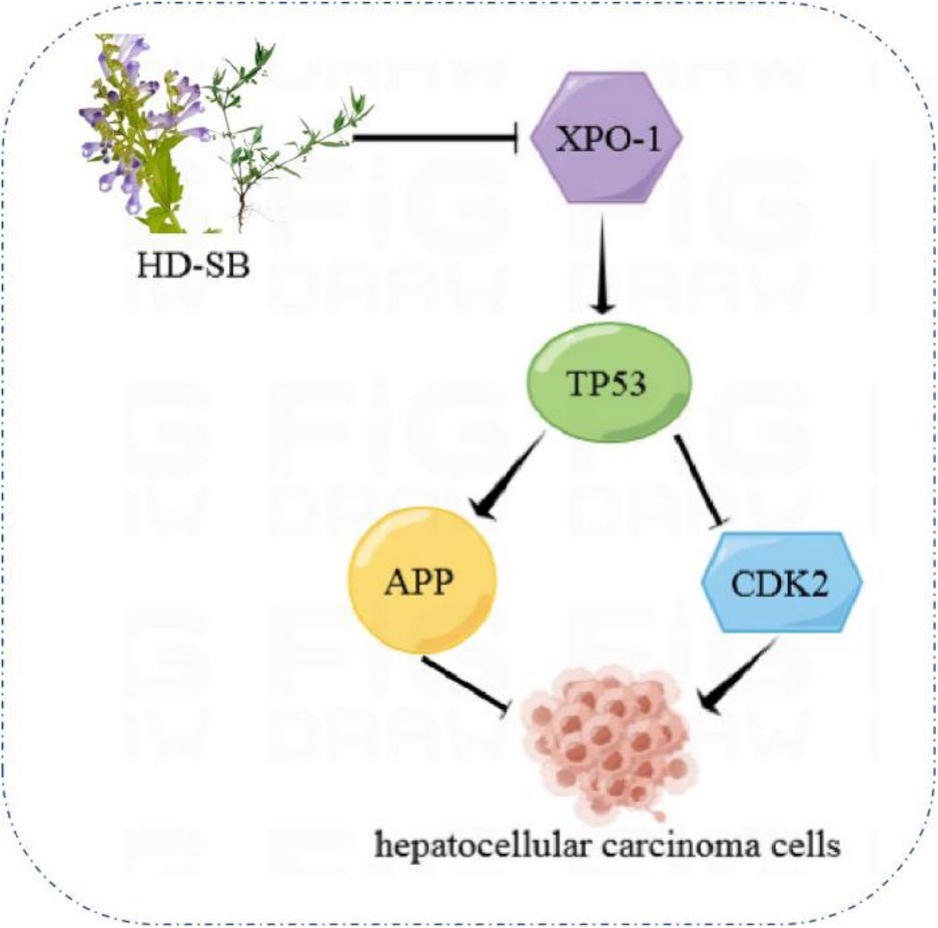
Schematic that illustrates the working principle of HD–SB-related signaling in HepG2 cells. We propose that HD–SB maintained XPO-1 expression, activating the TP53-dependent signaling pathway associated with HCC.

TP53 is an important oncogene, which can inhibit tumorigenesis by inducing cell cycle arrest or apoptosis. P53 can promote hepatocellular carcinoma cell apoptosis by increasing the expression of Bcl-2, P21, P14 and inhibiting the expression of BAX^29^. In this study, the functional analysis of GO and KEGG also found that the P53 signaling pathway was closely related to HD–SB for HCC. XPO1 is a nuclear transport receptor protein responsible for the nuclear export of some growth regulators and tumour suppressors. Studies have shown that inhibition of XPO1 expression can activate TP53 and induce cancer cell apoptosis^30^. APP expression is regulated by TP53 and induces apoptosis by inhibiting the mitochondrial apoptotic pathway and the expression of the apoptosis suppressor gene Bcl-2^31^. CDK2 belongs to the CDK family and can phosphorylate a large number of transcription factors, involved in the regulation of a variety of cancer signaling pathways, and promote cancer development. Studies have shown that CDK2 expression is associated with HCC size and TNM stage^32^. TP53 is involved in the regulation of CDK2 mRNA and protein expression levels^16^. Our results showed that the three active components of HD–SB increased P53 and APP levels and decreased XPO1 and CDK2 levels in vitro. Based on the above results, we propose that HD–SB up-regulates TP53 expression by inhibiting XPO-1, thus increasing APP and inhibiting CDK2 levels, and inhibiting HCC cell proliferation (Fig. 8).

In summary, this study used the network pharmacology and the molecular docking system to analyse the mechanism of action of HD–SB in the treatment of HCC, revealing that HD–SB acts on multiple targets to play a tumour suppressor role and the characteristics of HD–SB in the treatment of HCC through multiple components. Our study provides a theoretical basis for the treatment of tumours with HD–SB. Our subsequent studies will further study the specific mechanism of HD–SB regulation of liver cancer cells.

## Materials and methods

### Regents and materials

Dulbecco's modified eagle medium (DMEM), double antibody, foetal bovine serum (FBS), and pancreatic enzyme were obtained from GENOM (Hangzhou, Zhejiang province, China). Phosphate buffered saline (PBS) was obtained from Wuhan Servicebio Technology Co., Ltd. (Wuhan, Hubei, China). Cell counting Kit-8 (CCK-8) and dimethyl sulfoxide (DMSO) were obtained from Bio-sharp (Hefei, Anhui province, China). Purifications of quercetin, luteolin, beta-sitosterol, and baicalein were purchased from MedChemExpress (Shanghai, China).

### Network pharmacology

#### Target source of HD–SB and HCC

The HD–SB compounds were obtained from the Database of Traditional Chinese Medicines for Systems Pharmacology (TCMSP, https://tcmspw.com/tcmsp.php). Active compounds that meet the requirements are identified according to the screening conditions for oral bioavailability (OB) < 30% and drug similarity (DL) < 0.18. The target names of the compounds that met the requirements were recorded. HD–SB targets were collected using the TCMSP database. For compounds lacking targets, the PubChem database was used to search the compound ID number, the chemical structure of SMILES was queried for target complement through the SwissTarget Prediction database, and finally the obtained targets were converted into unified gene names using the Uniport database^33^. HCC-relevant targets were obtained by searching the keyword 'Hepatoma', 'Hepatic Carcinoma', or 'Hepatocellular Carcinoma' from the GeneCards public database (https://www.genecards.org/).

#### Data filtering and visual analysis

A Venn diagram was used to draw data on the intersection of HD–SB compound targets and disease targets. The protein–protein interaction (PPI) data was obtained from STRING online (https://string-db.org). The Bisogenet plug-in in Cytoscape3.8.2 software was used to analyse the PPI network for HD–SB HCC targets. Using CytoNCA plug-in to analyse topology parameters BC (Betweenness Centrality), CC (Closeness Centrality) and DC (Degree Centrality) in network, and screen key targets through topology parameters. The screening criterion is greater than the average value of DC, BC, and CC.

#### Enrichment analysis

Using the DAVID 6.8.0 database (https://david.Ncifcrf.gov/) and setting thresholds < 0.05, GO function and the enrichment analysis of the KEGG pathway^34,35^ of HCC targets treated with HD–SB were performed and the generated results were visualised.

#### Molecular docking technology

The main active components in the core target network diagram were selected for molecular docking with the key targets. PDB file for the target in the PDB database and SDF file for the active ingredient in the PubChem database. AutoDock Vina software was used for molecular docking and PyMOL software was used to visualise the results.

### Cell line and cell culture

The HepG2 human hepatocarcinoma cell line and the Huh7 cell line were purchased from the American Type Culture Collection (ATCC). Cells were cultured in DMEM complete medium containing 10% foetal bovine serum at 37° C and 5%CO_2_ and incubated in an incubator with saturated humidity. When the growth density of the cells reached 90%, subculture was carried out and the cells in the logarithmic growth stage were used for experimental research.

### Cell viability assay

Cell viability was evaluated using a cell count kit-8 assay (CCK-8) following the manufacturer's protocol. We seeded 2 × 10^3^ cells in 96-well plates and incubated at 37° C for 24 h. Cells were treated with various concentrations of quercetin, luteolin, baicalein, and beta-sitosterol for 24 h or 48 h, respectively, in a humidified chamber containing 5% CO_2_. Then we added 10 μl CCK-8 reagent to each well and incubated for 1.5 h at 37 °C. A microplate reader (Bio-Tech, USA) was used to determine the absorbance of cells at 450 nm (OD450). GraphPad Prim 9.0 software was adopted to calculate the value of 50% inhibitory concentration (IC50).

### Quantitative reverse transcription polymerase chain reaction

Gene expression was determined by quantitative reverse transcription polymerase chain reaction (qRT-PCR) analysis. Total RNA was isolated from HepG2 cells and Huh7 cells using the SteadyPure Universal RNA Extraction Kit (AG21017; Invitrogen), and then the 1 μg sample of RNA was reverse transcribed using a PrimeScriptTM RT Master Mix (Perfect Real Time) (RR036A; Takara, Shiga, Japan) according to the manufacturer’s instructions. Relative gene levels were determined by RT-qPCR using the StepOnePlusTM real-time PCR system (Applied Biosystems, USA). All RT-qPCR mixtures were prepared using a TB Green® Premix Ex Taq™ II (Tli RNaseH Plus) (RR820A; Takaka) with specific primers (Table 4). The mRNA levels of all target genes were normalized to the expression of the housekeeping gene actin. Relative quantities were determined using the comparative ΔΔCt method.

**Table 4:** RT-PCR primer sequence.

Gene	Primer sequence (5′-3′)	
TP53	Sense	GAGGTTGGCTCTGACTGTACC
	Antisense	TCCGTCCCAGTAGATTACCAC
XPO1	Sense	AGCAAAGAATGGCTCAAGAAGT
	Antisense	TATTCCTTCGCACTGGTTCCT
APP	Sense	TCTCGTTCCTGACAAGTGCAA
	Antisense	GCAAGTTGGTACTCTTCTCACTG
CDK2	Sense	CCAGGAGTTACTTCTATGCCTGA
	Antisense	TTCATCCAGGGGAGGTACAAC
β-actin	Sense	CGTAAAGACCTCTATGCCAACA
	Antisense	AGCCACCAATCCACACAGAG

### Western blot analysis

Cells from each group were lysed with RIPA lysate for 30 min and then transferred to the centrifuge tube. After centrifuging at 12,000 rpm/min for 10 min, the supernatant was extracted. Quantitative protein concentration was detected by the BCA Protein Assay Kit. After being separated by SDS-PAGE, 50 µg protein samples were transferred. onto the PVDF membrane, sealed with 5% skim milk powder at room temperature for 1 h, and then washed with TBST solution. Rabbit anti-P53, XPO1, APP, and CDK2 monoclonal antibodies (1:1000) were added, respectively, to incubate overnight at 4 °C, then the membrane was washed again, the corresponding secondary antibody was added, and the ECL kit was used to stain. The grey value of each imaging protein band was analysed by the Gel Imaging System, and the relative expression change of each group protein was compared with Beta actin as an internal reference.

### Statistical analysis

Data were expressed as mean ± SD. The results were analysed using GraphPad Prism 9.0 and SPSS 20.0 software. Student's t tests were developed to compare quantitative data between groups and *p* < 0.05 revealed a significant difference.

## Data Availability

All data generated or analysed during this study are included in this published article.
